# Pioneering family physicians and the mechanisms for strengthening primary health care in India—A qualitative descriptive study

**DOI:** 10.1371/journal.pgph.0001972

**Published:** 2023-06-08

**Authors:** Archna Gupta, Ramakrishna Prasad, Sunil Abraham, Nisanth Menon Nedungalaparambil, Megan Landes, Carolyn Steele Gray, Sanjeev Sridharan, Onil Bhattacharyya

**Affiliations:** 1 Department of Family and Community Medicine, St. Michaels Hospital, Toronto, Canada; 2 Department of Family and Community Medicine, University of Toronto, Toronto, Canada; 3 Institute of Health, Policy, Management and Evaluation, University of Toronto, Toronto, Canada; 4 PMCH Restore Health, Bangalore, Karnataka, India; 5 National Centre for Primary Care Research & Policy, Academy of Family Physicians of India (AFPI), New Delhi, India; 6 Department of Family Medicine, Christian Medical College, Vellore, Tamil Nadu, India; 7 Department of Emergency Medicine, AIMS, Thrissur, Kerala, India; 8 Bridgepoint Collaboratory for Research and Innovation, Lunenfeld-Tanenbaum Research Institute, Sinai Health System, Toronto, Ontario, Canada; 9 Health Policy Evaluation, Social Science Research Institute, University of Hawaii at Manoa, Honolulu, Hawaii, United States of America; University of Minnesota, UNITED STATES

## Abstract

India has one of the most unequal healthcare systems globally, lagging behind its economic development. Improved primary care and primary health care play an integral role in overcoming health disparities. Family medicine is a subset of primary care—delivered by family physicians, characterized by comprehensive, continuous, coordinated, collaborative, personal, family and community-oriented services—and may be able to fill these gaps. This research aims to understand the potential mechanisms by which family physicians can strengthen primary health care. In this qualitative descriptive study, we interviewed twenty family physicians, identified by purposeful and snowball sampling, who are among the first family physicians in India who received accredited certification in FM and were identified as pioneers of family medicine. We used the Contribution of Family Medicine to Strengthening Primary Health Care Framework to understand the potential mechanisms by which family medicine strengthens primary health care. Iterative inductive techniques were used for analysis. This research identifies multiple ways family physicians can strengthen primary health care in India. They are skilled primary care providers and support mid and low-level health care providers’ ongoing training and capacity building. They develop relationships with specialists, ensure appropriate referral systems are in place, and, when necessary, work with governments and organizations to access the essential resources needed to deliver care. They motivate the workforce and change how care is delivered by ensuring providers’ skills match the needs of communities and engage communities as partners in healthcare delivery. These findings highlight multiple mechanisms by which family physicians strengthen primary health care. Investments in postgraduate training in family medicine and integrating family physicians into the primary care sector, particularly the public sector, could address health disparities.

## Introduction

Primary health care (PHC) achieves health and well-being through primary care, public health, intersectoral policies, and empowering people and communities [1]. Strong PHC significantly reduces the leading causes of global morbidity and mortality, including maternal, neonatal, and child deaths from infections and vaccine-preventable diseases [2–4]. It reduces total healthcare costs, increases efficiency by improving access to preventative and promotive services, provides earlier diagnosis and treatment for many conditions, and delivers care that focuses on the whole person’s needs [5–8]. Primary care is an integral subset of PHC and concentrates on the health services provided to individuals [3, 9]. A robust PHC system plays a vital role in overcoming inequalities in health.

High-quality primary care describes services delivered to patients, including first-contact access for each new need, long-term person- (not disease) focused care, comprehensive care for most health needs, and coordinated care when sought elsewhere [5]. Family medicine (FM) is a subset of primary care delivered by family physicians—specialists in primary care. Family physicians are trained to provide person-centred, comprehensive and continuous care regardless of age, sex, and type of health problem, addressing most of the health needs of the communities they serve [10, 11]. In this research, family physicians are differentiated from general practitioners (GPs) by completing postgraduate training in FM, while GPs enter practice after completing medical school alone [12, 13].

Several countries are implementing FM to strengthen primary care and PHC. FM was introduced between the 1980s and the early 2000s in at least 21 lower and middle income countries (LMIC) [14]. To date, there is a lack of data to understand how FM is successfully implemented and the mechanisms by which FM strengthens PHC. By mechanism, we mean to describe what it is about FM implementation that brings about any effects [15]. Previous research has looked at macro level enablers of FM implementation in a country, but not how FM strengthens PHC [16–18]. We specifically look at the process of how individuals interpret and use FM to strengthen PHC in their local contexts [15]. FM implementation and its impact on PHC are challenging to evaluate, given that FM is a complex healthcare intervention interacting in complex social contexts [19]. A better understanding of these mechanisms could make current investments in FM in LMIC more effective.

India has a population of 1.42 billion people [20]–it is a large, diverse country, consisting of 28 states and eight union territories. Each has its own culture, many with its own language and, similarly, its own healthcare systems. It is an economically evolving nation, moving from a low-income country in 1990 to a middle-income country in 2010 [21]. Despite this economic advancement, the country still faces significant gaps in primary care and PHC. There has been a movement to introduce FM to strengthen PHC. Our associated article describes the historical evolution of PHC in India and how FM implementation may fill existing gaps [22]. This research aims to understand how family physicians strengthen PHC in the Indian context.

### Family medicine in India

Traditionally, GPs delivered primary care. These are individuals with an undergraduate medical degree—Bachelor of Medicine and a Bachelor of Surgery (MBBS)—equivalent to an undergraduate MD degree in Canada or the US. However, there has been a decline in the number of practicing GPs and concerns around their competence and capacity to deliver comprehensive primary care [23–25]. There have been concerns about the declining number of medical graduates choosing to work as GPs and instead choosing sub-specialty practice [25, 26]. This is partly due to the lack of academic recognition of general practice [27, 28]. Simultaneously, there are concerns about the capacity of GPs to provide quality care without additional postgraduate training [29]. The implementation of FM is viewed as a way of establishing a specialty cadre of physicians equipped and motivated to deliver primary care services and work with non-physician health care workers to increase the capacity of the primary care workforce. India’s medical education system also trains Community Medicine physicians, who are distinct from family physicians, both in training and practice. The focus of community medicine training is to produce medical professionals with expertise in public health and applied epidemiology and is viewed as an academic discipline supporting health system planning, research and policy and service delivery in academic settings [30].

FM implementation, defined by implementing postgraduate training and certification in FM, began in the 1980s. First, through a small movement of individuals who attained certification in FM after their MBBS. They were referred to as Members of the National Academy of Medical Sciences (MNAMs)–Family Medicine and achieved certification through continuing medical education, experience and passing a written and practical examination. Over time, the MNAMs accreditation program was converted to a government organization called the National Board of Examinations (NBE). FM was first recognized as a distinct specialty by the Medical Council of India (MCI) in 1984. Despite this recognition, FM’s postgraduate residency training programs did not appear until the early 1990s. First, Diplomate of National Board (DNB) programs awarded by the NBE started, leading to a Diplomate of National Board Family Medicine (DNB-FM) degree. In 2000, FM was included as a qualification awarded under the MCI itself, leading to a Doctor of Medicine in Family Medicine (MD-FM) [31]. The first MD-FM program started in 2012 [31]. MD programs occur in National Medical Commission (NMC) (which replaced the MCI in September 2020) accredited university teaching hospitals, while the NBE runs DNB programs parallel in non-teaching and private sector hospitals. In addition to full-time DNB-FM and MD-FM training programs, there are several part-time, distance-based or blended FM programs which neither the DNB nor NMC recognize. In the blended programs, participants receive distance teaching complemented with in-person teaching sessions. Distance and blended programs include the: (1)Post Graduate Diploma in FM; (2) Master in FM; (3) Diploma in FM; and (4) Fellowship in FM [31]. Additionally, several Indian medical graduates opt to train in FM through the Membership of the Royal College of General Practitioners (MRCGP) International program, where studying and completing an examination meets the United Kingdom’s international RCGP standards and confers membership [32].

Thirty-nine accredited private institutions offered the DNB-FM program out of a total of 276 as of 2023 [33, 34]. Five government medical colleges of a total of 286 offer the MD-FM program as of 2021 [33]. Over the last four decades, there have been multiple routes introduced to gain postgraduate training in FM. However, only full-time DNB and MD programs are accredited through the national regulatory boards (NBE and NMC).

### Aim

Since the introduction of FM in India, little research has been done to understand the impact of postgraduate training, leaving uncertainty about its value. Our study had two broad aims: (1) to understand how FM is implemented in a national context and the implementation trajectory, and (2) to understand how FM implementation influences PHC system strengthening. To address these aims, we had two research questions: (1) What are the potential mechanisms by which early cohort family physicians drive and sustain FM implementation in India and over what trajectory? and (2) What are the potential mechanisms by which early cohort family physicians strengthen PHC in India?

This paper describes the potential mechanisms by which early cohort family physicians strengthen PHC in India. Given that there are two different research questions and the volume of data, this study is reported in two articles. Our associated article describes how early cohort family physicians supported the emergence and implementation of FM in India [22].

## Materials and methods

### Ethics

This study was reviewed and accepted by the Research Ethics Board at the University of Toronto, Canada and from the Institutional Review Board (Health) at the Swami Vivekananda Youth Movement, India. Written consent was obtained from participants before the interviews. Consistent with participatory approaches, participants were given a choice to be identified by name or anonymously, enabling and empowering them to retain ownership of their stories [35].

### Study design

A participatory action research approach which applies qualitative description (QD) collection and analysis methods was used [36, 37]. The research questions were born from discussions with Indian family physician collaborators who challenged that one could not understand the emergence of FM in India without hearing the pioneers’ stories. As one of our collaborators put it: “If you do not listen to these stories, you will miss out a lot on the pioneering work for FM that happened in India”(SA). Indian family physician collaborators (RK, SA, NM) were involved in the research design, including defining the questions, designing the research protocol, and participating as research participants (SA). Action research engages professional social researchers with local stakeholders in a co-generative process of knowledge creation, action design, and outcomes evaluation [37]. Participation in action research is not just a “moral value” but essential because of the complexities of the problems addressed, ensuring participant knowledge is used to inform research practice [36].

Applying a QD collection and analysis approach was appropriate for this type of exploratory inquiry in which there is limited pre-existing research. QD is also appropriate to help describe and understand the underlying processes of FM implementation and PHC strengthening from the perspectives of participants [38–41]. In QD, researchers are actively involved in the research process where their understanding of the phenomena, in this case, FM, are seen as integral for new inquiry [38, 42, 43]. This was important for our study given that several authors involved in this study are family physicians themselves (AG, RK, SA, NM, OB, ML) and in the Indian context (RK, SA, NM). Using QD also allowed us to remain faithful to the participants’ accounts [43, 44].

### Participant recruitment

We identified FM pioneers in Tamil Nadu, Karnataka and Kerala, including early cohort family physicians, who received the designation through the MNAMS certification route or completed a three year full-time Diplomate of the National Board (DNB) postgraduate training in Family Medicine, or were teachers of the early cohort family physicians. These states were selected as they were the first to have introduced postgraduate training in FM and have the greatest number of training programs. Initially, purposeful sampling was used to identify participants known to have contributed to the field of FM [45, 46]. Participants were identified by Indian family physician collaborators and their networks. As the study progressed, snowball sampling was used [45], by asking participants to suggest others they would recommend for this study at the end of each interview.

### Data collection

To answer our research questions, we aimed to interview fifteen to twenty participants to allow for information-rich and detailed cases permitting thick descriptions [38, 47, 48]. Given that QD analysis emphasizes each participant’s individual experience and story’s uniqueness, data saturation can never truly be achieved and was not used as a parameter for determining sample size [38, 49]. We focused on a size that allowed us to adequately explore and answer the research question [38].

In-depth interviews were conducted. A semi-structured interview guide was used. Interview guides were pilot tested before their use with two Indian family physician research collaborators (RP, NM) to ensure question clarity and reduce redundancy [50]. Interviews were conducted in English by the principal author (AG) and were audio-recorded and transcribed verbatim. Interviews ranged from 40 minutes to 75 minutes. All interviews took place between August and October 2019 with one to two daily interviews.

Supplementary field notes and reflective memos were written after each interview to capture observations about the participants’ settings and geographic context, allowing the interviewer (AG) to immerse herself in the environment and to put participant stories into context, understanding how, where and to whom participants delivered care [51, 52]. Reflective memos allowed the interviewer (AG) to document reflections and perspectives on individual interviews, create a synopsis of each conversation, and note the data’s interpretation throughout the process, remaining attentive to what was unique or common across interviews.

### Data analysis

Interview field notes and memos were reviewed after each interview (AG). Researchers (AG and RP) discussed emerging themes throughout the three-month interview process, allowing early insights to be incorporated into ongoing data collection [52]. Transcription of interviews was done in two sets, first after interviews that were conducted in Tamil Nadu and then again after interviews were completed in Karnataka and Kerala. “Strategic periods of immersion in the field” occurred when the interviewer (AG) was actively interviewing participants in Southern India. This was “interspersed with periods of immersion in the data,” which supported testing and developing conceptualizations about the roles that early pioneers played in defining and implementing FM in India [53]. After repeated immersion in the data and “making sense of the data,” the transcripts were coded to identify categories, themes and linkage in the data and explore relationships and patterns between the data sources [38, 43]. Iterative, inductive analysis techniques were applied. Inductively identified codes were mapped onto the implementation frameworks described earlier.

A sample of six interviews was read, re-read and hand-coded to develop a codebook with emerging themes and subthemes [38]. The codebook was validated with a peer researcher. AG and the peer researcher independently read and coded three diverse transcripts (state of training, gender of participant, type of work) and compared findings to ensure consensus on the definition of codes. The codebook and a summary of the initial findings were sent to all participants for feedback. We received a response from 50% of the participants interviewed and no changes were made to the codebook. After hearing back from participants, all twenty transcripts were then re-read and hand coded. Analytic notes were taken during this process to document emerging connections and patterns within and between themes. All interviews were coded again, allowing for further reflection and organization of codes and for digitization of hand codes in NVivo 12 Plus [54].

### Conceptual framework

**Fig 1:**
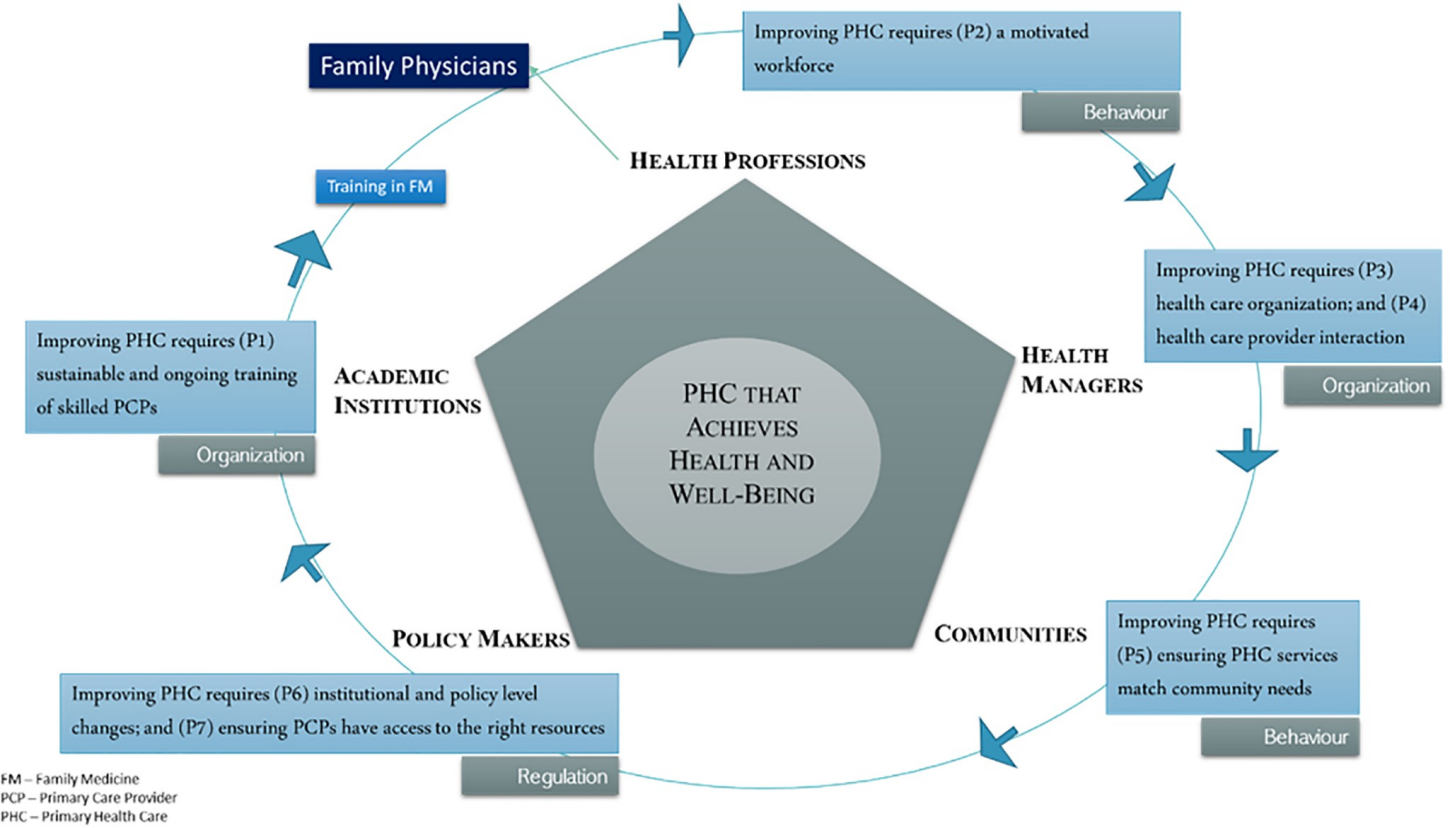
Contribution of family medicine to strengthening primary health care framework.

We applied the Contribution of Family Medicine to Strengthening Primary Health Care Framework (Fig 1) to guide the interview guide and analysis [14]. This framework provides sensitizing concepts to understand the mechanisms by which FM strengthens PHC. As defined by Patton, “sensitizing concepts are terms, phrases, labels, and constructs that invite inquiry into what they mean to people in the setting(s) being studied [55]"

In this framework, we propose that family physicians strengthen PHC by bridging the gaps between patients and health resources, individual health and public health, communities and academic medical centers, and primary care providers and specialists while working in harmony with other key stakeholders [12, 56, 57]. Family physicians influence the organization and regulation of healthcare and the behaviour of patients and providers to achieve health system changes [58]. The proposition statements highlight specifically how family physicians can support strengthening PHC (Table 1).

**Table 1:** Definitions for contribution of family medicine to strengthening primary health care framework.

Area of Influence	Definition		Proposition Statement
**Organization**	Mechanisms that affect the mix of providers in healthcare systems, their roles and functions and how providers operate, thus determining the kinds of provider organizations that exist and their structures	**P1**	Improving PHC requires sustainable training of skilled generalist primary care providers (PCPs) (physicians and non-physicians)
		**P3**	Improving PHC requires changes in how care is organized and delivered and how organizations interact with one another
		**P4**	Improving PHC requires changes in how health care providers interact with one another
**Behaviour**	Efforts to influence how individuals act with health and healthcare, including patients and providers.	**P2**	Improving PHC requires a motivated workforce of PCPs
		**P5**	Improving PHC requires health care providers to work with communities to ensure services offered match community needs
**Regulation**	The use of policy to alter the behaviour of actors in the health system	**P6**	Improving PHC requires institutional and policy level changes that support prioritizing primary care
		**P7**	Improving PHC requires PCPs have access to the right resources

In this study, we focus on how family physicians motivate a workforce of future family physicians and non-physician primary care providers; change how healthcare is organized and delivered; ensure primary care services meet community needs; change how health care providers interact with one another and ensure primary care providers have access to the right resources. We do not specifically speak to the sustainable training of skilled primary care providers and policy and institutional changes, as these conditions were described in the associated article [22].

## Results

### Sample description

Twenty participants were interviewed (Table 2). Eight participants were female. Eight participants were from Tamil Nadu, six from Karnataka and six from Kerala. Two participants were physicians trained in internal medicine, one of whom switched to FM practice–and completed postgraduate FM training- early in his career, becoming one of the first educators of FM in India. The second was a strong advocate of FM training and became the first MD FM program department head. Three participants were physicians who received Member of National Academy of Medical Sciences (MNAMS)–Family Medicine certification in the early 1980s through continuing medical education and completing a certification exam before the implementation of full-time postgraduate training programs. Fifteen participants were early cohort graduates of full-time DNB-FM postgraduate training programs in either Tamil Nadu, Karnataka, or Kerala. Eight participants primarily work in rural or remote communities, six in urban settings, and nine in academic training institutions.

**Table 2:** Participant characteristics.

**Current State of Practice** *	
Tamil Nadu	8
Karnataka	6
Kerala	6
**Family Medicine Certification**	
MNAMS-FM	3
International FM Degree	1
DNB-FM	15
None	1
**Current Location of Practice** **	
Rural	7
Urban	6
Academic	7
**Gender**	
Female	8
Male	12

*State of practice does not necessarily reflect the state of training.

**Some participants had exposure to multiple settings and were included under the setting they practiced in at the time of the interview.

We found that implementing FM training strengthened PHC by creating a cadre of skilled primary care providers. Additionally, through the actions of early cohort family physicians, FM strengthened PHC by contributing to a motivated workforce, promoting healthcare organization and delivery changes, collaborating with communities to promote primary care services meeting their needs, improving how healthcare providers interact and advocating for access to the right resources.

### Motivated workforce

We sought to describe how early cohort family physicians influence a motivated workforce (proposition 2). Not only did these individuals choose to pursue a profession in primary care, a sector that has historically been neglected and undervalued in India, but they continue to deliver care to patients in this complex environment and forge new paths for themselves and future family physicians. This article focuses on how early cohort family physicians motivate others in the field. A description of what motivated these individuals to enter the field is reported separately [22].

From the data, what was overwhelmingly heard was how family physicians are mentors for others. Many early cohort family physicians described feeling inspired and engaged by mentors and went on to mentor future trainees and graduates themselves. Dr. Rajkumar, a pioneering family physician who is believed to have implemented the first FM postgraduate training program in India, shares how he perceives the specialty of FM, its importance and how he imparts this message to his trainees (Table 3, Quote 1). Early cohort family physicians also share the impact mentors have had on themselves and how they see it as their role to inspire future FM learners (Table 3, Quotes 2, 3, and 4). FM resonated with the personal values of these participants, which may have contributed to their motivation and ability to appeal to others with similar values.

**Table 3:** Motivated workforce participant quotes.

Quote Number	Quote	Participant
1	“I think FM, as often said, is more than just a specialty. It is a mission in itself of trying to understand and empower people, give them choices and respect them and allow patient-centered care. That’s unique, and it is interesting how that uniqueness makes it possible to cope with all the theory and knowledge they have from the specialist’s point. But at the same time, make sure they understood what they need to know. It takes a lot of time, at least a year, for a person to realize what they are trained for. The trainees initially did not know what they had come to train for. So you need to help them to say that now you are doing something special, unique, you should be proud of it, so that was the key thing that is special to train people for, and that was very challenging.”	Dr. Rajkumar Ramasamy, Tamil Nadu
2	“I am proud to say that I am a student of Dr. Rajkumar who…was very highly motivated to provide primary care in rural communities and he is very passionate about FM, and he has been a mentor and role model to us … He wrote a book on primary care for health workers and for doctors … One of his students, Dr. Sunil Abraham, now he is heading the FM Department in CMC Vellore and few of the students who graduated from Oddanchatram, we have been involved in different forms of primary care, so he has been very influential and has been influencing us for primary care even till today.”	Dr. Isac David, Tamil Nadu
3	“Dr. B.C. Rao, in Bangalore he is, I would say, one exemplary family physician person who has always been working towards the betterment. I will quote him also. He also keeps telling ‘I didn’t have the support, but now when I am with you all, I feel like we can do it.’ …He has been a good mentor for all of us.”	Dr. Swapna Bhaskar, Karnataka
4	“We need champions in FM to take up the cause. So when our residents go there, they will train them well. Fifteen years back, we did not have champions.”	Dr. Prince Christopher, Tamil Nadu

### Skilled primary care providers

An essential component of strengthening PHC is ensuring that primary care has skilled providers (proposition 1). Five main themes emerged from what participants felt they gained from postgraduate training in FM. First, they described increased confidence to address patients’ needs (Table 4, Quotes 5 and 6). Second, they gained practical hands-on skills, including procedural skills, which they did not experience during their undergraduate MBBS studies allowing them to address the broad scope of patient issues seen in primary care. Dr. Swapna highlights that focused postgraduate training in FM allows for greater training opportunities (Table 4, Quote 7). Dr. Bhaskara and Dr. Venkatesh shared examples of how the procedural skills they learned allow them to manage the care of patients in the primary care sector in rural and remote contexts (Table 4, Quotes 8 and 9). Dr. Serin, who worked as a GP before completing postgraduate training in FM, compares his ability to care for patients as a GP versus a family physician (Table 4, Quote 10). Third, participants portrayed learning about the importance of patient-centeredness (Table 4, Quote 11). Fourth, several participants spoke about the beneficial impact of educational experiences in rural communities during their postgraduate training. These placements allowed family physicians to realize the impact they could have on addressing the community’s needs (Table 4, Quotes 12 and 13). Finally, multiple participants underscored how working in rural and community placements during their FM training also provided them with the opportunity to work with non-physician healthcare workers as a part of a team. Through these experiences, they also learned how to be involved with capacity building and teaching of non-physician healthcare workers (Table 4, Quotes 14 and 15).

**Table 4:** Skills gained from postgraduate training in family medicine quotes.

Quote Number	Quote	Participant
Increased Confidence		
5	“I became very confident. My practice looked very simple because I knew so much more than a general practitioner knew.”	Dr. SR Jayaprakash, Karnataka
6	“My training and the confidence, what I got in doing the skills also, even in this small setup I used to do the earlobe repair, even small procedures like suturing, the I&Ds [incisions and drainage] and all that we do… my diagnosis system will be different, and the procedures I involve and do will be different, and the referral will be definitely different, so that is the strength.”	Dr. Ally Plackal, Kerala
Increased Practical and Procedural Skills		
7	“Unlike a medical college where there are one hundred people, so there is a whole lot of difference [in postgraduate FM training] where you get basically hands-on, on every procedure on the patient itself. One to one interaction with the patient. One to one interaction with your mentor. Which doesn’t happen in undergraduates.”	Dr. Swapna Bhaskar, Karnataka
8	“Almost all the things which can be done in a hospital setting they taught us to do even on a solo basis. In the sense, for example, starting from simply putting an IV cannula, simple tube catheterization, any CSF [cerebral spinal fluid] analysis, lumbar puncture and pleural fluid aspiration for diagnostic and therapeutic purpose, ascitic fluid tapping and foreign body removal from either ear, nose, or any other things and small excisions and minority procedures … almost all the things I am doing in my practice now.”	Dr. Bhaskara Puttarajanna, Karnataka
9	“I have managed some challenging things which I would not have managed without this [FM] training. When I went for [my] first [FM training] rotation, I could not diagnose an ectopic pregnancy …But, at the end of the training, I could manage a young boy with a bomb blast. He handled a bomb while playing, without knowing it was a bomb, kept for digging wells. So, he came in with multiple injuries, bleeding and pellets all over the body. So, I could give him sedation, started a line, resuscitated him, gave him morphine and removed all the foreign bodies from his body and transported him in an ambulance.”	Dr. Venkatesh S., Tamil Nadu
10	I had worked before post-graduation and after post-graduation. So basically [before postgraduate FM training] it was just like [I was] a traffic police standing in the middle of a junction, so I was feeling like……when a patient comes with a chest pain you have to direct that patient to the cardiologist when a person comes with a wheeze you have to direct that person to the pulmonologist … so basically I was feeling like we are good for nothing … after [postgraduate FM training] I realized that actually, we can manage, I would say more than 90%, up to 95%, because the reference [referral] rate is very minimal, not even 5%, from my experience. So that was the major difference… I am confident to do all those things, not only the non-surgical part, even the surgical part.	Dr. Serin Kuriakose, Kerala
Increased Capacity to Deliver Patient Centered Care		
11	“I learnt a lot of skills. I learnt to think through problems. I learnt what good medicine is. I learnt to be sensitive to the patient’s economic realities, social realities, which though it was taught as theory in MBBS, I saw it actually. Now, here is a patient from a village who is poor. We were taught you might be able to offer the gold standard treatment, but if the patient can’t afford it, there is no point, so we were made to think like that. So, that made a difference and understanding the biopsychosocial model of medicine.”	Dr. Sunil Abraham, Tamil Nadu
Increased Capacity to Understand and Address Community Needs		
12	“That was a place where we developed our communication skills. At least some of the principles of FM, like not looking at their disease, it was like seeing their old prescriptions, avoiding the polypharmacy…How to manage those patients, how to spend time with them, how to understand what their real problems are, all these things this community posting only gave that.”	Dr. Resmi S. Kaimal, Kerala
13	“I built relationships. I got to know the community. I mapped them. I could identify which were the problem houses, and we did a lot of home visits. We did a lot of health education apart from the clinics. So that gave me a very good foundation for primary care work.”	Dr. Vijila Isac, Tamil Nadu
Increased Capacity to work with and Train Interdisciplinary Team Members		
14	“[During training] we were not exactly training health workers formally, but since we had nursing students like that, so we learned the methodology of teaching health workers which came in very helpful when we went to Jharkhand because there the entire system depended on training community health workers.”	Dr. Isac David, Tamil Nadu
15	“The other thing that this nurse and I did was we identified health workers in all these villages and started the training. So, I think that is why they needed this solid four months. So we would call them back to the health centre. They will spend the whole day there. We will do the training in various fields, like how to identify fevers and the different types of fevers…The idea being that they would also visit schools and teach there even when we are not there and sanitation, so this started the training program for the health workers as well.”	Dr. Malini Devanandan, Tamil Nadu

### Healthcare organization and delivery changes

Changing how healthcare is organized includes changing who delivers care and their roles in service delivery (proposition 3). FM is a field where family physicians exercise their professional role by providing care directly to patients or working collaboratively with other health professionals based on the health needs and resources available in the communities in which they work [12, 59].

Dr. Isac and Vijila are believed to be the first two graduates of FM training in India. Following postgraduate FM training, they married and moved to Jharkhand, previously a part of Bihar. There they implemented a primary care system, and later a secondary care system, for an isolated tribal group, known as the Malto people, a population of 100,000 who did not previously have access to healthcare. A key to their success was building capacity in the community through training local community health volunteers who were integral to the primary care system.

Dr. Vijila describes the context of the region in which they chose to work, highlighting the geographic challenges for delivering care in this setting (Table 5, Quote 16). They realized several potential barriers to successfully delivering care to the region’s population and worked to mitigate them. First, two family physicians could not have direct contact with the entire population. Second, community involvement was essential to ensure engagement and support. To address these challenges, they created a system for training community health volunteers from the villages they were serving (Table 5, Quote 17). They developed and implemented creative training methods to educate community members, first about health prevention, identifying health illnesses, and then identifying diseases and dispensing medications (Table 5, Quote 18).

**Table 5:** Healthcare organization and delivery changes participant quotes.

Quote Number	Quote	Participant
16	“They used to have epidemics of cholera. Lots and lots of malaria throughout the year. Epidemics of kala-azar, pneumonia, childhood deaths. It was all infectious diseases mainly and maternal issues. So, when we saw the need, we finally decided to go there. And we just started with a small team of five, Isac and myself, a nurse, a lab technician and a pharmacist, and we started from a single room clinic. The first thing we realized was because you can’t even call them villages, they were hamlets, an average hamlet would have like 15 houses…they were scattered all over the hills. Most of the villages didn’t have road access…It was not possible to have direct physical access…The only way possible was to train community health workers.”	Dr. Vijila Isac, Tamil Nadu
17	“We need to identify the health problems and identify the local people who can be trained to take care of their own health needs. So we formulated a vision that we will improve the health status of the community by empowering the people.”	Dr. Isac David, Tamil Nadu
18	“We selected community health volunteers from each village … We met these volunteers once a month for training, and initially it was like three days we would go to their village, stay with them and train… 80% were illiterate, which meant that we could not use the traditional methods … We had to start using a lot of innovation in our teaching… We started writing songs for them for each health problems. There is a song for malaria, a song for diarrhea, a song for kala-azar, even for a topic like delivery or infertility, we had songs…That made a huge difference. So totally illiterate men and women were able to learn these songs. And after some months once they were confident about the different common health problems and because of the very high mortality, we had to take a decision to give them the medicines.”	Dr. Vijila Isac, Tamil Nadu
19	“Because we were just the two of us, and we also had to manage the clinical work and administration and the training and the community work. The only way to do it [start a hospital] was to train nurse practitioners, which we did all through those 20 years. I believe the nurses are well able to handle at least 80% of what a doctor can… I believe that they are, in fact, the most important part of a team and equipping them like teaching them and empowering them to take a lot of decisions and identify the red flags when a doctor really needs to see a patient. By doing that, I realized we could achieve a lot-lot more than if we had tried to see every patient.”	Dr. Vijila Isac, Tamil Nadu
20	“After a couple of years, we worked in remote areas. We reached a point where we again and again and again saw the importance of training, and so actually we asked that hospital where we worked, ‘can we also train’…But the next two hospitals I worked at was really busy, and I was the only doctor. There was no way I can say, like if I have a group of health workers to train and there is this lady who is having eclamptic convulsions, and in [the operating] theatre, I can’t say, like I have to teach them or I have to do that urgent thing, but I know that lady won’t be there if these people are trained, and they can control her hypertension in the village, she won’t have come into the eclamptic stage. So that’s the hard thing.”	Dr. Jachin Velavan, Tamil Nadu
21	“We also developed … the one-year course for laypersons, it is not for doctors but for people who are in the remote areas can be trained in this, that program has a 60-day hands-on because they really have to know the skills.”	Dr. Jachin Velavan, Tamil Nadu
22	“We increased our outreach… with the different staff like the health workers, the outreach nurses … and we want to go out more on the streets where the poorest of the poor live who won’t come to the hospital…”	Dr. Sunil Abraham, Tamil Nadu

After eight years in the region, they had successfully built a functioning primary care system consisting of two family physicians and a network of community health volunteers from each community. However, they realized their work was not complete. To continue to meet the community’s needs, they also needed to establish a system for patients to access secondary care. To do this, they built a thirty-bed hospital. Again, they understood that the two of them alone could not directly see and treat every patient. They needed to train a team of non-physician health care providers to work with them as a part of a team model. They achieved this through training nurse practitioners to deliver secondary care in the hospital alongside themselves (Table 5, Quote 19). They spent the first twenty years of their FM careers in this community in northern India before moving back to their native state, Tamil Nadu.

The theme of family physicians building capacity among non-physician health care providers to address the rural communities’ needs was seen again when speaking to Dr. Jachin. She spent the first half of her career working as a solo family physician in Bihar, Odisha, and Assam’s remote communities. She shared feeling conflicted, wanting to spend more time with patients and addressing primary and preventative health but could not do more than “episodic or curative” care due to the high demand. She negotiated with hospitals to train community health workers but continued to feel the tension between her role as an educator and a healthcare provider (Table 5, Quote 20). Given her experiences working in rural and remote areas and understanding the benefit of education programs to increase the skills of GPs and other non-physician primary care providers, she relocated back to the Christian Medical College (CMC) in Vellore Tamil Nadu. Here, she helped develop and expand the Postgraduate Degree Program in FM (PGDFM), a distance-based course for FM training targeting GPs, and developed training education programs for mid-level and low-level non-physician primary care providers (Table 5, Quote 21).

The idea of family physicians building outreach programs and training community health volunteers from communities was also seen among academic family physicians working in urban settings. At CMC Vellore in Tamil Nadu, they realized that the number of vulnerable patients seeking care from their clinics declined. To ensure they received the necessary care, family physicians developed a Community Oriented Primary Care Program (COPC), where physicians train and work with community health workers and community health volunteers (Table 5, Quote 22). They created a registry of patients and delivered care through mobile clinics to informal urban settlements, focusing on providing continuity of care to patients identified with chronic diseases.

These examples show how early cohort graduates challenge healthcare system norms such as how health care should be delivered and by whom and focused on training non-physician health care workers to support primary and secondary care delivery alongside family physicians.

### Promoting primary care services to meet community needs

The previous section highlights the motivation for service delivery changes to promote delivering healthcare that meets the communities’ and patients’ needs. This section speaks specifically of how early cohort family physicians engage communities as partners in healthcare (proposition 5).

Dr. Rajkumar describes the primary care system he and his wife developed in KC Patti, a small hill station in Tamil Nadu. They serve a population of 15,000, most of whom are tribal people or agricultural workers in remote villages and account for some of the most neglected communities in the state. He highlights the importance of community engagement and involvement to ensure appropriate services and that patients feel respected (Table 6, Quote 23). Dr. Vijila describes how they would work with the families and communities, listen to their stories to understand their health problems and develop innovative education tools based on patient stories to help provide preventative counselling that would resonate with the community (Table 6, Quote 24).

**Table 6:** Promoting primary care services to meet community needs participant quotes.

Quote Number	Quote	Participant
23	“We now have a system of primary care where we have a base health centre with people trained from the most vulnerable sections of the community, which is crucial to our work because they represent people. They tell us what is feasible, what is not. And also, people from the vulnerable sections feel more at home and respected when they come here because they know their own people are working here.”	Dr. Rajkumar Ramasamy, Tamil Nadu
24	“For example, immunization, we had visited this village where I think three or four kids had died of measles. So, we listened to how it actually happened. And then we built a story around that, and then according to the story, you get the volunteers to do a skit, take photographs, and that is converted into flashcards… So, through the songs and the skits and flashcards quickly the health awareness started to improve.”	Dr. Vijila Isac, Tamil Nadu

Including patients and community members in the delivery of health care services and the design of development of health education tools was critical for the work of these family physicians.

### Improving how healthcare providers interact

Improving PHC requires changes in how healthcare providers interact with one another (proposition 4). From the previous sections, we see examples of family physicians training and collaborating with non-physician healthcare providers. This section looks specifically at how family physicians develop networks with specialists as part of referral pathways or support for their patients. Family physicians are generalist specialists and often need to collaborate with discipline-specific specialist physicians when patients need care or procedures outside their scope.

Dr. Vijila shared how, when working in rural contexts, they had to seek out and build relationships with specialists in the region for referral and help support them remotely to care for their patients (Table 7, Quote 25). This ability to broker these types of relationships is central to the practice of FM. Dr. Resmi, an academic family physician, shares that collaborating with specialists is just as integral in urban contexts. As FM is an emerging field, some tensions exist, akin to “turf wars,” where providers may defend their interests over the patient’s interest. She highlights the importance of her role in being flexible in finding ways to collaborate with specialists as FM evolves as a field and finds its place in the health system (Table 7, Quote 26).

**Table 7:** Improving how healthcare providers interact participant quotes.

Quote Number	Quote	Participant
25	“I developed a good network of doctors that I could talk to, and that helped a lot. So like a patient might have a problem, an endocrinological problem, but I could always contact a couple of endocrinologists who could sort it out for me. And out there in the remote village, I was able to handle it.”	Dr. Vijila Isac, Tamil Nadu
26	“We should remember is that everyone is insecure in this field now. So let us not fight with them [specialists], we have to be a little bit flexible…We are not here to grab the patients.”	Dr. Resmi S. Kaimal, Kerala

Brokering relationships with non-physician primary care providers and specialist physicians appears to be vital to the work of early cohort family physicians. Particularly in the Indian context, where FM is an emerging field where many are unaware of FM, the role of family physicians, and how they should interact with one another. Family physicians play a significant role in developing these relationships.

### Gaining access to the right resources

Promoting high-quality PHC requires having access to the right resources (proposition 7). From our participants who developed primary care systems in rural contexts, it was evident that collaborations were critical to access the necessary equipment and material resources needed to deliver care.

For example, Dr. Isaac shares their experience of delivering preventive measures such as immunizations and mosquito nets to the population. They were only able to do this by accessing resources from the government. However, to be able to do this required advocacy and persistence on their part (Table 8, Quote 27 and 28). Alternatively, Dr. Rajkumar shared that collaborating with the government was not always possible as the community’s needs were at odds with the government’s guidelines (Table 8, Quote 29). This led family physicians to find other innovative collaborations with non-governmental organizations to serve their communities.

**Table 8:** Gaining access to the right resources participant quotes.

Quote Number	Quote	Participant
27	“We wanted to do immunization services. We were willing to do immunization at the village. But vaccines had to be provided by the government. But here [we are], two young doctors with no credibility of their own going to start a community health program. The district administration was very skeptical. So I had to go to the district headquarters seven times, which means a whole day of visit, going on a two-wheeler. So by the seventh visit, the district collector understood our work because we were there for almost one to one and half years, and they started seeing our work, and the district collector approved for the first batch of vaccines. Then the district medical officer gave the vaccines.”	Dr. Isac David, Tamil Nadu
28	“We were able to make an impact in reducing the incidence of kala-azar, and when it came to malaria, then the government started coming to us because in the tribal communities, it was difficult to dispense mosquito nets. So when we approached the district authorities, at one go they gave 5000 mosquito nets to be distributed in tribal villages and eventually they also gave us pyrethroid and DDT insecticide for spraying in the villages… They partnered with us for control programs.”	Dr. Isac David, Tamil Nadu
29	“The government, we tried working with, and we have probably been guilty of not trying to go back and work with them in the last few years. But in the initial stages, their concept of primary care was difficult, and when you approach them that you could work with them, they laid down fairly rigid rules which clearly would not be what would be in the best interest of practicing FM. They are more interested in audits and data collection and things, whereas the practical need was to win a relationship with the community and set up a primary care system that was treating people.”	Dr. Rajkumar Ramasamy, Tamil Nadu

## Discussion

Our research highlights multiple mechanisms by which FM and family physicians potentially strengthen primary care and PHC in India which are congruent with the proposed conceptual framework (Fig 1). They are skilled primary care providers and support mid and low-level health care providers’ ongoing training and capacity building. They develop relationships with specialists, ensure appropriate referral systems are in place, and, when necessary, work with governments and organizations to access the essential resources needed to deliver care. They motivate the workforce and change how care is delivered by ensuring providers’ skills match the needs of communities and engage communities as partners in healthcare delivery.

Our findings are consistent with research from other regions globally highlighting how postgraduate training in FM increases the ability to deliver primary care as compared to general practitioners (with undergraduate MBBS training alone) (P1). Family physicians feel postgraduate training in FM enhances their ability to deliver primary care compared to general practitioners–those with undergraduate MBBS training alone. They are more confident and have a greater breadth of skills, including procedural skills. This helps some feel more comfortable working in smaller remote communities where tertiary care access is limited. Our findings are similar to other studies globally. A pre-and post-survey assessed the perceived confidence of trainees for clinical skills before and after postgraduate FM training in Gezira State in Sudan and found significant increases in confidence in performing procedural skills post FM training [60]. Similarly, research done in Canada, just after the implementation of postgraduate training in FM and before it was mandatory, found that those who had completed postgraduate FM training compared to those who had not scored higher on evaluation indicators including charting, procedures and quality of medical care [61]. Although our research did not directly assess patient perspectives, participants perceived their patients appreciated their level of care, evidenced by their willingness to seek out care from family physicians. These findings are similar to those of from cross-sectional study from Thailand, where patients perceived family physicians offer better communication skills, improved continuity of care and enablement (the patient’s knowledge of a self-care plan after the consultation) compared to GPs [62]. Our research also adds how they feel better equipped to provide patient-centred care, communicate more effectively, and assess the needs of their communities. Additionally, continuity of care among a patient panel–an ongoing relationship with a defined group of patients over time–is considered a core component of primary care in many regions. This appeared to be addressed in training programs which emphasized patient-centred care and community and rural placements where trainees visited the same communities and families over time. We see that family physicians found ways to incorporate this into their practice through their development of Community Oriented Primary Care (COPC) programs for defined populations. This study highlights that postgraduate training in FM increases the capacity to deliver primary care, however, to achieve sustainable training government support is necessary to increase training opportunities in the field and to create pathways for family physicians to be integrated into the existing healthcare system [22].

Not only did our participants feel that they increased their capacity to deliver care from FM training, but they learned how to work with and train non-physician healthcare workers as a part of a team model (P1). Family physicians provide ongoing training and capacity building to their non-physician team members and tailor their services, including the mix of health care providers, to meet the community’s needs. Our participant stories showed how they did just this in both rural and urban contexts. These findings are not unique to India. A scoping review of FM in sub-Saharan Africa also found that family physicians play essential roles in mentoring and training supportive health care staff as part of team models [18]. Similarly, In Chile, family physicians are described as playing an indispensable role as advisors to team members as part of interdisciplinary teams caring for patients and families [63]. In Brazil, family physicians were added to family health program teams to complement the skills of other health workers and improve the technical skills of team members [12, 64]. Globally, research shows that family physicians play essential roles in mentoring and training supportive health care staff as part of interdisciplinary team models. Our research highlights how incorporating exposure to interdisciplinary teams in FM training is valuable.

This research also adds to the literature by providing examples of how family physicians create a motivated primary care workforce through their direct engagement with individuals and indirectly through their work (P2). Health worker motivation—the extent to which an individual is willing to exert and maintain an effort towards achieving organizational goals—is a critical barrier to effective health service delivery and contributor to human resources shortages [65–67]. Participant stories also underscore how family physicians recruited members from their communities and trained them over the years. More importantly, they shared how they viewed them as integral team members, shifting away from authoritative leadership styles. This is evident through the stories where participants speak about "empowering" community health volunteers and ensuring a relationship based on "mutual respect." Research from India highlights the importance of staff motivation in the successful implementation of PHC services and also retention and performance of healthcare workers [68, 69].

Several participants described how they changed how care was delivered for geographically defined communities working to address patients’ care needs both from a public health and primary care perspective (P3). This was seen in both rural and urban contexts. Family physicians first assessed the community’s health needs and then determined programs and services to be delivered. This feature of family physicians developing and providing COPC is also seen in research from Sub-Saharan Africa [18, 70]. Our research found that community engagement was a critical facilitator of service delivery changes, specifically the implementation of COPC programs (P5). Family physicians designed and implemented programs with input from beneficiaries. Community members were often brought in as active and ongoing team members of the COPC program as trained community health volunteers or workers where continuous bi-directional learning can be achieved. Although our research identifies how family physicians changed how care was delivered within their own institutions and regions, what was evident was their limited capacity to successfully spread these changes to a state or national level without government support.

Family physicians brokered relationships with other specialist physicians to deliver care for their patients (P4). Collaboration between these groups is critical for ensuring continuity and coordination between primary, secondary, and tertiary care [71, 72]. Our associated study and other global research has found that tension between these groups is prevalent in India and countries where FM is emerging [17, 22, 73]. There may be a lack of understanding of how different providers can effectively work with one another. This research highlights how pioneering family physicians overcome these tensions and developed relationships and referral pathways with specialists.

Family physicians collaborate with system level stakeholders to ensure access to the right health care resources in settings with limited primary care infrastructure (P7). For pioneering family physicians developing primary care systems in remote regions or underserved populations in urban contexts, they ensured they had access to resources, whether medications, vaccines or supplies, equipment, funds and even facilities, was essential. Alternatively, sometimes individuals felt they could not access what they needed from governments and sought out what they needed from non-governmental organizations. These inputs into healthcare are widely accepted as being critical for well-functioning healthcare systems [74]. In countries such as India, where FM appears to emerge from a "bottom-up" approach, where individual leaders drive FM implementation as opposed to policy it is apparent the vital role pioneering family physicians play in sourcing the necessary inputs for service delivery [22, 75, 76]. Although this was a feasible approach for pioneering family physicians in discrete geographic regions, government support is critical as the field of FM grows, to ensure populations have access to the right resources.

The stories highlighted in this research are unique in that several pioneering family physicians in India were visionaries who built new primary care systems, serving large populations. While they have demonstrated it is possible, it is not clear how many will follow them. This is likely to be only one of several paths to strengthening PHC. Yet, the experience of these family physicians does highlight multiple mechanisms by which FM could strengthen PHC. However, government support for FM is an essential component for implementation that achieves institutionalization in the country–whereby FM is an integral part of primary care delivery [22].

### Strengths and limitations

This research was done using a participatory action research approach where participants were involved in the study design, implementation and analysis allowing for rich recruitment of participants and deep understanding of complex mechanisms. Our sample represented family physicians working in urban, rural and academic settings and explored the implementation of FM in three unique States. This diverse representation supports transferability of findings to similar settings and regions. This study used several techniques to promote rigour and credibility [48]. Rigour was ensured by researcher immersion in the field, and memoing and producing field notes. Credibility was sought using codebook validation with a peer researcher and member checking. Member reflections were also integrated into the codebook. Multivocality was achieved by ensuring participatory research methods and having participants as research collaborators.

This study is also subject to several limitations. Recent research has shown that primary care providers in the Southern States, including MBBS graduates (GPs) and informal untrained workers, have greater medical knowledge than those in the Northern States [24]. This research included participants from three Southern States; however, their work did take them outside of their states and to Northern India, as described in participant stories. Future research could look at the role of family physicians across northern states.

This research only focused on family physicians and did not include non-physician participants or patients. We speculate from participant stories that family physicians contribute to health care worker motivation and capacity building. Including non-physician health care workers working with family physicians would be helpful to test these hypotheses and bring forward other insights about the strength of these mechanisms. Including patients could help garner whether they perceive a different quality of care when received directly by a family physician or a team led by a family physician versus GPs or teams made of solely non-physician primary care providers. Finally, including community members and health system managers could provide validation to the health system benefits of FM.

## Conclusion

Our research highlights the various mechanisms by which the implementation of FM could potentially strengthen PHC in India. Family physicians are specialist physicians who deliver primary and secondary care services, motivate others to work in the field, manage health care teams, and change how care is provided, prioritizing communities’ needs. Investing in postgraduate FM training and integrating family physicians as an integral part of primary care teams could improve primary care and PHC.
